# Oropharyngeal Dysphagia in Dermatomyositis: Associations with Clinical and Laboratory Features Including Autoantibodies

**DOI:** 10.1371/journal.pone.0154746

**Published:** 2016-05-11

**Authors:** Naoki Mugii, Minoru Hasegawa, Takashi Matsushita, Yasuhito Hamaguchi, Sacihe Oohata, Hirokazu Okita, Tetsutarou Yahata, Fujiko Someya, Katsumi Inoue, Shigeyuki Murono, Manabu Fujimoto, Kazuhiko Takehara

**Affiliations:** 1 Department of Rehabilitation, Kanazawa University Hospital, Kanazawa, Ishikawa, Japan; 2 Department of Dermatology, Division of Medicine, Faculty of Medical Sciences, University of Fukui, Yoshida-gun, Fukui, Japan; 3 Department of Dermatology, Faculty of Medicine, Institute of Medical, Pharmaceutical and Health Sciences, Kanazawa University, Kanazawa, Ishikawa, Japan; 4 Division of Rehabilitation Science, Faculty of Medicine, Institute of Medical, Pharmaceutical and Health Sciences, Kanazawa University, Kanazawa, Ishikawa, Japan; 5 Department of Otorhinolaryngology/Head and Neck Surgery, Faculty of Medicine, Institute of Medical, Pharmaceutical and Health Sciences, Kanazawa University, Kanazawa, Ishikawa, Japan; 6 Department of Dermatology, Faculty of Medicine, University of Tsukuba, Tsukuba, Ibaraki, Japan; Nippon Medical School Graduate School of Medicine, JAPAN

## Abstract

**Objective:**

Dysphagia develops with low frequency in patients with dermatomyositis. Our objective was to determine the clinical and laboratory features that can estimate the development of dysphagia in dermatomyositis.

**Methods:**

This study included 92 Japanese patients with adult-onset dermatomyositis. The associations between dysphagia and clinical and laboratory features including disease-specific autoantibodies determined by immunoprecipitation assays were analyzed.

**Results:**

Videofluoroscopy swallow study (VFSS) was performed for all patients with clinical dysphagia (n = 13, 14.1%) but not for patients without clinical dysphagia. Typical findings of dysphagia (pharyngeal pooling, n = 11 and/or nasal regurgitation, n = 4) was detected by VFSS in all patients with clinical dysphagia. Eleven patients with dysphagia (84.6%) had anti-transcription intermediary factor 1γ (TIF-1γ) antibody. By univariate analysis, the average age and the male to female ratio, internal malignancy, and anti-TIF-1γ antibody were significantly higher and the frequency of interstitial lung diseases and manual muscle testing (MMT) scores of sternomastoid and dertoid muscles were significantly lower in patients with dysphagia than in patients without dysphagia. Among patients with anti-TIF-1γ antibody, the mean age, the ratios of male to female and internal malignancy were significantly higher and mean MMT scores of sternomastoid muscle were significantly lower in patients with dysphagia compared with patients without dysphagia. By multivariable analysis, the risk of dysphagia was strongly associated with the existence of internal malignancy and ant-TIF-1γ antibody and was also associated with reduced scores of manual muscle test of sternomastoid muscle. Dysphagia was markedly improved after the treatment against myositis in all 13 patients.

**Conclusion:**

These findings indicate that dysphagia can develop frequently in patients with internal malignancy, anti-TIF-1γ antibody, or severe muscle weakness of sternomastoid muscle.

## Introduction

Polymyositis (PM) and dermatomyositis (DM) are autoimmune connective tissue diseases characterized by symmetric proximal myositis. PM and DM are similar diseases, but DM can be distinguished from PM via the presence of specific skin lesion such as heliotrope rash or Gottron’s papule/sign. Clinical manifestations of DM are heterogeneous and interstitial pneumonia and internal malignancy affect the prognosis.

Dysphagia has been reported to develop in 10 to 73% of patients with inflammatory myopathy during the clinical course, especially in inclusion body myositis [1–3]. The dysphagia in this disease primarily affects the skeletal muscle-activated oropharyngeal phase of swallowing. This may occur due to weakness of oropharyngeal, laryngeal, and esophageal musculature. Recognition of dysphagia is important, since dysphagia is associated with various clinical features such as nasal speech, hoarseness, regurgitation, nutritious deficits, aspiration pneumonia, impaired quality of life, and poor prognosis. It has been reported that dysphagia involvement is more frequent in patients with internal malignancy among patients with DM [4, 5].

Recent studies demonstrated that myositis-specific autoantibodies are associated with specific clinical phenotype [6]. Among them, antibodies against a 155-kd protein (anti-p155 Ab) and a 155/140-kd doublet (anti-155/140 Ab) have shown a significant association with malignancy in adult patients with DM [7–9]. Transcription intermediary factor 1γ (TIF-1γ) and TIF-1α/γ have been considered to be the autoantigen target of these Abs, respectively [10]. A patient with DM who was positive for anti-TIF-1γ Ab had 27-fold higher odds of having malignancy compared with a patient who was negative for the Abs [11]. On the other hand, pulmonary involvement is frequently seen in patients with anti-aminoacyl transfer RNA synthetase (ARS) Abs [12] or anti-melanoma differentiation-associated protein 5 (MDA-5) Ab [13, 14]. DM patients with anti-Mi-2 Abs typically do not have interstitial lung disease (ILD) or internal malignancy and have a good prognosis [15]. However, the association between autoantibodies and the involvement of dysphagia remains unclear. Furthermore, to our best knowledge, almost no studies have analyzed the associated clinical or laboratory features focused on dysphagia in DM.

In this study, we assessed the association between the oropharyngeal involvement and clinical or laboratory features including recently identified autoantibodies.

## Materials and Methods

### Patients

Ninety-two Japanese patients with DM (72 females and 20 males; age (mean ± SD), 54.9 ± 16.2 years) who visited Kanazawa University Hospital between January, 2004 and December, 2015 were included in this study. Eighty patients fulfilled the criteria of Bohan and Peter [16, 17], while the remaining 12 did not fulfil the criteria, but fulfilled Sontheimer’s criteria [18], due to the absence of clinical muscle symptoms and presence of subsistent clinical skin eruptions. Mean disease duration was 9.7 ± 14.9 months. At the time of evaluation, no patients were receiving oral prednisolone therapy and other immunosuppressive drugs.

### Clinical assessment

Complete medical histories, physical examinations, and laboratory tests were conducted for all patients during the first visit, with limited evaluations during follow-up visits. The objective symptoms of pharyngeal pooling, nasal regurgitation, and nasal speech were evaluated by otorhinolaryngologists and speech therapists. The patients were diagnosed as having ILD according to the results of chest radiography, chest computed tomography, and pulmonary function testing. Serum Krebs von den Lungen-6 (KL-6) levels as a serum marker of ILD were determined by ELISA as described previously [19]. Presence of internal malignancy was carefully examined using computed tomography, gastrointestinal fiberscope, gallium scintigraphy and other procedures according to need. The medical ethics committee of Kanazawa University approved this study protocol (No. 961, An investigation of rehabilitation for dermatomyositis/polymyositis). An informed written consent was obtained from each patient.

### Videofluoroscopy swallow study (VFSS)

The existence of dysphagia was confirmed by VFSS in patients with clinical dysphagia. A fluoroscopy unit (Siemens Polystar digital unit- Siemens AG, Erlangen, Germany) connected to a medical high definition medical quality videorecorder was used. Videofluoroscopy studies were performed in a lateral position with the patient in a seated position. The patient was asked to swallow a food and/or liquid bolus of high density (EZPaque 100% wpv) barium sulphate suspension. Images were acquired at 15 pulses/s.

### Myositis disease activity assessment

To assess disease activity based on individual organ systems, the Myositis Disease Activity Assessment Visual Analogue Scales (MYOACT VAS) portion of the Myositis Disease Activity Tool [20] was used. The MYOACT assessment utilizes separate 100-mm VAS to gauge the physician’s evaluation of disease activity in several discrete domains. Involvement of all nonmuscle organ systems (constitutional, cardiac, pulmonary, gastrointestinal, skeletal, and cutaneous) was also evaluated using the composite extra-skeletal muscle VAS score. An additional VAS measure, the global VAS score, was used to rate overall disease activity.

### Immunoprecipitation assays

Immunoprecipitation assays were performed to identify autoantibodies using extracts of the leukemia cell line, K562, as previously described [13]. Using this method, 23 of 92 patients were positive for anti-ARS Ab, 26 patients were positive for anti-TIF-1γ Ab, 3 patients were positive for anti-Mi-2 Ab, 15 patients were positive for anti-MDA-5 Ab, 5 patients were positive for anti-SS-A (Ro60) Ab, 5 patients were positive for anti-U1 ribonucleoprotein (U1 RNP) Ab, and a patient was positive for anti-signal recognition particle (SRP) Ab. Four patients were positive both for anti-ARS Ab and anti-SS-A Ab. One patient was positive both for anti-MDA-5 Ab and anti-U1 RNP Ab.

### Statistical analysis

Statistical analysis was performed using Student’s t test or Fisher’s exact probability test. The relationship between the involvement of dysphagia and explanatory variables was analysed using multivariable analysis (logistic regression). Significant variables after univariate regression (p<0.15) were entered into a stepwise multivariate model. Results are expressed as the OR with 95% CI. A p-value <0.05 was considered indicative of statistical significance.

## Results

### Videofluoroscopy swallow study (VFSS)

**Fig 1:**
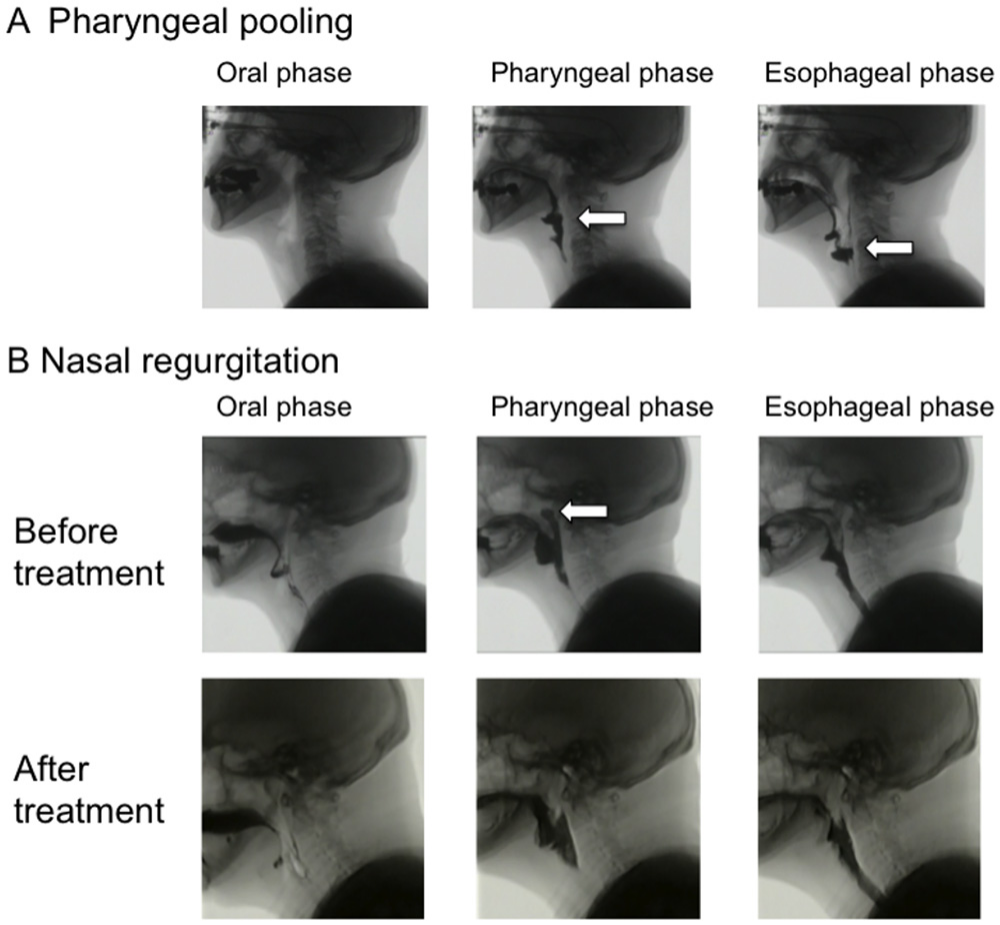
Videofluoroscopy swallow study (VFSS). Videoradiographic sequence in a patient with dermatomyositis (DM). A) Pharyngeal pooling is detected at both pharyngeal and esophageal phases. An arrow indicates abnormal pooling of imaging agent. B) Nasal regurgitation is detected at pharyngeal phases before treatment of DM. However, the observation was disappeared after treatment with corticosteroid. An arrow indicates nasal regurgitation of imaging agent.

We considered that 13 patients had clinical dysphagia by history taking and examination of otolaryngologists and speech therapists. To confirm the existence of dysphagia, VFSS was performed in these 13 patients and all patients showed the typical findings of dysphagia (pharyngeal pooling; n = 11 and/or nasal regurgitation; n = 4). Dysphagia was found at the pharyngeal phases, but not at oral stage or esophageal stage in all of these patients. A representative case of pharyngeal pooling is shown as Fig 1A and S1 Fig. VFSS of a representative case of nasal pooling was also shown before and after treatment of DM (Fig 1B and S2 Fig). Before treatment, she showed nasal regurgitation at pharyngeal phases. This patient also developed nasal speech, probably due to the loss of pharyngo-oesophageal muscle tone. However, nasal regurgitation detected by VFSS and nasal speech were diminished after oral treatment of 40 mg/d prednisolone (S2 Fig). VFSS could not perform in any patients without clinical dysphagia.

### Association between dysphagia involvement and clinical/laboratory features

Firstly, we examined the association between the frequency of dysphagia involvement and clinical or laboratory features by univariate analysis (Table 1). Most remarkable association was detected between dysphagia and the existence of anti-TIF-1γ Ab or internal malignancy. Most patients with dysphagia had internal malignancy (84.6%) or anti-TIF-1γ Ab (84.6%). And this frequency was significantly higher than that of patients without them (10.1% and 19.0%, respectively, p<0.0001). Age and the frequency of male/female ratio were significantly higher in patients with dysphagia compared with patients without dysphagia (p<0.01). The manual muscle testing (MMT) of neck (sternomastoid muscle) and shoulder (dertoid muscle) but not hip (iliopsous muscle) was significantly lower in patients with dysphagia compared with patients without dysphagia (p<0.05). The frequency of ILD was significantly lower in patients with dysphagia compared with patients without it (p<0.01). Otherwise, there were no significant differences between the frequencies of any clinical/laboratory features. Thus, dysphagia was significantly more detected in aged or male patients, patients with internal malignancy, anti-TIF-1γ Ab, and reduced MMT scores of sternomastoid and dertoid muscles or patients without ILD.

**Table 1:** Associations between dysphagia and clinical or laboratory features in patients with dermatomyositis.

	Dysphagia + (n = 13)	Dysphagia − (n = 79)
Age	67.8±10.2**	52.8±16.1
Sex (male:female)	7: 6**	13: 66
Disease duration (month)	3.9±4.8	10.6±15.8
Symptom		
Muscle weakness	92.3%	68.4%
MMT (sternomastoid)	2.8±1.1*	4.0±1.2
MMT (deltoid)	3.5±1.2*	4.3±0.9
MMT (iIliopsous)	3.7±0.9	4.1±0.9
Gottron's sign	92.3%	67.9%
Heliotrope rash	53.8%	43.6%
Interstitial lung diseases	7.7%**	52.6%
Internal malignancy	84.6%***	10.1%
Laboratory finding		
Elevated CK	92.3%	54.4%
Elevated KL-6	23.1%	47.4%
Autoantibodies		
Anti-ARS antibody	7.7%	27.8%
Anti-TIF-1γ antibody	84.6%***	19.0%
Anti-Mi-2 antibody	0%	3.8%
Anti-MDA-5 antibody	0%	19.0%
Anti-SS-A (Ro60) antibody	0%	6.3%
Anti-U1 RNP antibody	0%	6.3%
Anti-SRP antibody	0%	1.3%

MMT, manual muscle testing; CK, creatine kinase; KL-6, Krebs von den Lungen-6; ARS, aminoacyl-tRNA synthetase; TIF-1γ, transcription intermediary factor 1γ; MDA-5, melanoma differentiation-associated protein 5; RNP, ribonucleoprotein; SRP, signal recognition particle.

*p<0.05

**p<0.01

***p<0.0001 vs. patients without dysphagia

### Association between dysphagia involvement and clinical/laboratory features in patients with anti-TIF-1γ Ab

We also evaluated the difference of clinical/laboratory features between patients with or without dysphagia in DM patients with anti-TIF-1γ Ab (Table 2). The mean age and the ratios of male to female and internal malignancy were significantly higher in patients with dysphagia compared with patients without dysphagia (p<0.05, p<0.05, and p<0.001, respectively). On the other hand, the mean MMT scores of sternomastoid muscle were significantly lower in patients with dysphagia compared with patients without dysphagia (p<0.05). Other clinical/laboratory features were not significantly different between 2 groups.

**Table 2:** Associations between dysphagia and clinical or laboratory features in dermatomyositis patients with anti-TIF-1γ antibody.

	Dysphagia + (n = 11)	Dysphagia − (n = 15)
Age	66.8±9.9*	54.7±19.0
Sex (male:female)	6: 5*	1: 14
Disease duration (month)	4.4±5.1	5.0±6.1
Symptom		
Muscle weakness	90.9%	53.3%
MMT (sternomastoid)	2.9±1.2*	4.0±1.2
MMT (deltoid)	3.6±1.0	4.3±1.5
MMT (iIliopsous)	3.7±1.0	4.2±1.0
Gottron's sign	81.8%	73.3%
Heliotrope rash	72.7%	80.0%
Interstitial lung diseases	0%	6.7%
Internal malignancy	90.9%**	20.0%
Laboratory finding		
Elevated CK	90.9%	60.0%
Elevated KL-6	20.0%	20.0%

TIF-1, transcription intermediary factor 1; MMT, manual muscle testing; CK, creatine kinase; KL-6, Krebs von den Lungen-6.

*p<0.05

**p<0.001 vs. patients without dysphagia

### Independent factors that were associated with the development of dysphagia

Next, we evaluated clinical or laboratory factors that could closely associated with the development of dysphagia in patients with DM by multivariate analysis. Investigated factors were as follows; age, gender, interstitial lung diseases, internal malignancy, elevated CK, MMT scores (sternomastoid, deltoid, and iliopsous muscle), anti-ARS Ab, and anti-TIF-1γ Ab. We performed logistic regression analysis and the final model confirmed the significant risk factors for the development of dysphagia in DM; internal malignancy (odds ratio 22.2), anti- TIF-1γ Ab (odds ratio 11.8), and MMT score of sternomastoid muscle (odds ratio 0.46 for each score, Table 3).

**Table 3:** Multivariate analysis assessing the existence of dysphagia.

	OR (95% CI)	p value
Internal malignancy	22.2 (3.84 to 200.4)	0.0014
Anti-TIF-1γ Ab	11.8 (1.87 to 113.3)	0.0142
MMT (sternomastoid)	0.46 (0.20 to 0.95) for each score	0.0487

TIF-1, transcription intermediary factor 1; MMT, manual muscle testing

### The profile of patients with dysphagia

Based on these findings, the profile of 13 patients with dysphagia was evaluated (Table 4). Ten patients had both anti-TIF-1γ Ab and internal malignancy. Case 2 (56 year-old, female) was positive for anti-TIF-1γ Ab but did not have internal malignancy. Case 4 (64 year-old, male) with lung cancer was negative for anti-TIF-1γ Ab and positive for one kind of anti-ARS Ab (anti-OJ Ab). Another case with dysphagia (case 11; 83 year-old, female) was negative for known autoantibodies by immunoprecipitation assay and did not have malignancy. All patients with dysphagia had muscle weakness and/or elevated serum CK levels. The sequence of symptoms such as skin, muscle, and dysphagia was not fixed in each patient. All 13 patients showed clinical improvement of dysphagia after treatment of malignancy (n = 10) and DM (corticosteroid therapy; n = 13 and intravenous immunoglobulin therapy; n = 2) during 7.3±5.5 months period. Swallowing rehabilitation and balloon dilation were concurrently used in 11 cases and 1 case, respectively.

**Table 4:** The profile of dermatomyositis patients with dysphagia.

							MMT								
Case	Age	Sex	Duration (months)	Dysphagia	Autoantibody	Internal malignancy	Sternomastoid	Deltoid	Iliopsous	Elevated CK (IU/l)	Heliotrope rash	Gottron's sign	ILD	Treatment (maximam dose)	Sequence of symptoms
1	48	Female	3	Nasal regurgitation	Anti-TIF-1γ	Ovary	3	4	4	十 (418)	十	十	一	PSL 30mg, DR	Skin➡ Muscle, dysphagia
2	56	Female	3	Pharyngeal pooling	Anti-TIF-1γ	none	2	3+	2-	十 (270)	十	十	一	PSL 60mg, mPSL pulse, DR	Skin➡ Muscle, dysphagia
3	56	Male	18	Pharyngeal pooling	Anti-TIF-1γ	Upper pharynx	ND	ND	ND	十 (262)	十	十	一	PSL 30mg	Skin➡ Muscle, dysphagia
4	64	Male	2	Pharyngeal Pooling, Cricopharyngeadysfunction, Nasal regurgitation	Anti-OJ	Lung	2-	2-	2	十 (2808)	十	一	一	PSL 20mg, mPSL pulse, IVIG,DR, B	Muscle➡ dysphagia➡ Skin
5	70	Male	8	Pharyngeal pooling, (Laryngeal elevation)	Anti-TIF-1γ	Stomach	2	2	3	十 (3084)	十	十	一	PSL 50mg, mPSL pulse, DR	Skin➡ Muscle, dysphagia
6	72	Male	2	Pharyngeal pooling	Anti-TIF-1γ	Sigmoid colon	3	4	4	十 (9560)	一	十	一	PSL 50mg, mPSL pulse, DR	dysphagia➡ Skin➡ Muscle
7	74	Female	2	Pharyngeal pooling, Nasal regurgitation	Anti-TIF-1γ	Gallbladder	2	3-	2	十 (2804)	一	十	一	PSL 50mg, DR	Skin, Muscle, dysphagia
8	75	Male	7	Pharyngeal pooling	Anti-TIF-1γ	Lung	3-	4+	4	一 (92)	十	十	一	PSL 50mg	Skin➡ Muscle, dysphagia
9	76	Female	1	Nasal regurgitation	Anti-TIF-1γ	Ovary	2-	2	2	十 (957)	十	十	一	PSL 40mg,IVIG, DR	Skin➡ Muscle, dysphagia
10	79	Male	1	Pharyngeal pooling	Anti-TIF-1γ	Lung	4	5	4	十 (1704)	一	十	一	PSL 30mg, DR	Skin, Muscle, dysphagia
11	83	Female	1	Pharyngeal pooling	None	none	4	5	5	十 (358)	一	十	十	PSL 30mg, DR	Skin➡ dysphagia ➡ Muscle
12	64	Male	2	Pharyngeal pooling	Anti-TIF-1γ	Lung	5	4	4	十 (537)	一	十	一	PSL 30mg, DR	Skin➡ Muscle➡ dysphagia
13	65	Female	1	Pharyngeal pooling	Anti-TIF-1γ	Breast	2	2	5	十 (637)	一	十	一	PSL 50mg, DR	Skin, Muscle, dysphagia

ILD, interstitial lung diseases; TIF-1, transcription intermediary factor 1; ND, not determined; MMT, manual muscle testing; CK, creatine kinase; PSL, prednisolone; mPSL, methyl PSL; CyA, cyclosporin A; IVIG, intravenous immunoglobulin; DR, dysphagia rehabilitation; B, balloning

Among 10 patients with both anti-TIF-1γ Ab and internal malignancy, internal malignancy was treated by both surgical operation and chemotherapy in 6 cases. The malignancy was treated with surgical operation, chemotherapy, and radiation in each one case and another one case could not receive any therapy for the malignancy. Only three cases achieved complete remission of the malignancy.

### Association between dysphagia and disease activity

To assess disease activity based on individual organ systems, MYOACT portion of the Myositis Disease Activity Assessment Tool was used. The VAS scales of muscle disease activity, global disease activity score, but not global extra-skeletal muscle disease activity score were significantly higher in patients with dysphagia compared with patients without it (p<0.01, Table 5). Among global extra-skeletal muscle disease activity, the VAS scales of cutaneous disease activity score in addition to gastrointestinal disease activity score were significantly elevated in patients with dysphagia compared with patients without it (p<0.05 and p<0.001, respectively). Thus, patients with dysphagia showed higher muscle, cutaneous and global disease activity of DM.

**Table 5:** Associations between dysphagia and myositis disease activity scale.

	Dysphagia (n = 13)	No dysphagia (n = 79)	p value
Muscle disease activity	35.4 ± 23.8	14.4 ± 17.5	<0.01
Global extra-skeletal muscle disease activity	17.8 ± 9.9	12.2 ± 6.8	ns
Constitutional disease activity	24.2 ± 23.1	12.3 ± 13.7	ns
Cutaneous disease activity	50.5 ± 19.9	30.5 ± 21.2	<0.05
Skeletal disease activity	1.0 ± 2.8	3.7 ± 7.7	ns
Gastrointestinal disease activity	11.9 ± 22.1	1.1 ± 3.7	<0.001
Pulmonary disease activity	8.1 ± 13.5	18.3 ± 20.9	ns
Cardiac disease activity	0.8 ± 2.8	1.5 ± 4.5	ns
Global disease activity	32.9 ± 17.5	18.4 ± 12.3	<0.01

## Discussion

In this study, we detected a most significant association between the involvement of dysphagia and the existence of internal malignancy or anti-TIF-1γ Ab. In addition, reduced MMT of sternomastoid muscles was independent risk factor for the involvement of dysphagia.

There are literatures that have demonstrated the increased frequency of dysphagia in DM patients with internal malignancy compared with patients without malignancy [4]. A recent Japanese study demonstrated that proximal dysphagia, older age, and absence of interstitial lung disease were independent risk factors for malignancy in Japanese patients with DM or PM [5]. In consistent with these previous studies, the involvement of dysphagia was positively associated with the involvement of internal malignancy by multiple regression in our population.

A recent systematic review and metaanalysis demonstrated that adult DM patients who were positive for anti-TIF-1γ Ab had 27-fold higher odds ratio of having malignancy [11]. Therefore, the increased frequency of internal malignancy may be due to the secondary tendency due to the existence of anti-TIF-1γ Ab in our study. Alternatively, the association between anti-TIF-1γ Ab and dysphagia may be due to the correlation between internal malignancy and dysphagia. In either case, our findings suggest that the existence of anti-TIF-1γ Ab is one of the best indicators to estimate the development of dysphagia in adult patients with DM, although previous studies did not evaluate this Ab. However, there is a phenotypic difference dependent on the age at onset among patients with this Ab. For example, juvenile patients with this Ab do not develop malignancy [7, 21]. In fact, the mean age and ratios of male to female were significantly higher in patients with dysphagia compared with patients without dysphagia among patients with anti-TIF-1γ Ab (Table 2). Although multivariate analysis did not define the aging or sex as an explanatory variable for the existence of dysphagia, further large studies may be needed to clarify the significance of age or sex for the development of dysphagia. Thus, patients with either internal malignancy or anti-TIF-1γ Ab may develop dysphagia more frequently compared with other patients.

A previous study demonstrated that the majority of DM patients with autoantibody against small ubiquitin-like modifier activating enzyme develop cutaneous disease and progressed to myositis with systemic features including dysphagia [22] The frequency has been reported as 8% (9/131 cases) in United Kingdom Caucasian adult-onset DM patients and the frequency of dysphagia has been detected in 7 of 9 patients. This Ab was also detected in 1.5% (7/456 cases) of Japanese DM patients and two of seven with this Ab had dysphagia [23].

Most patients with anti-TIF-1γ Ab exhibit DM with typical skin lesion and myositis, without ILD [7, 8]. Our findings including MYOACT assessment also indicate that dysphagia involvement tended to be associated with disease activity including global, muscle and skin in addition to gastrointestinal tract in patients with DM. All 11 patients with dysphagia had elevated CK levels and/or muscle weakness. However, a previous study demonstrated that clinical assessment of muscle strength or disease activity can not be used to predict the risk of swallow dysphagia in DM [24]. Our findings suggest that dysphagia develops in associated with multiple factors. Therefore, it is possible that dysphagia may be found during the active phase of DM if the patients had risk factors such as internal malignancy and/or anti-TIF-1γ Ab. Interestingly, reduced muscle strength of sternomastoid muscles but not other muscles was independent risk factor for the involvement of dysphagia, although sternomastoid muscles are close to the muscle of swallowing. Furthermore, the clinical symptoms of dysphagia disappeared after the therapy against DM in all 13 patients. In a representative patient, VFSS was very useful to observe the symptoms of dysphagia and to evaluate the effect of treatment.

The frequency of dysphagia was low in our cohort when compared with previous reports. We assessed the existence at the presentation, although previous reports may include the development of dysphagia during long clinical course. The definition of dysphagia may also different, because we evaluated it by otorhinolaryngologists and speech therapists and confirmed using VFSS. The mechanism of dysphagia of DM remains unclear. A recent study using VFSS and manometry indicated that dysphagia in inflammatory muscle diseases may be due to impaired muscle contraction and reduced hyolaryngeal excursion than the often held belief of failed upper esophageal sphincter relaxation [25].

In our study, the number of patients, especially patients with dysphagia is small. Further longitudinal studies in a larger population will be needed to clarify the associated factors for the development of dysphagia in patients with DM.

## Supporting Information

S1 Fig Moving image of Fig 1 (case 1 of Table 4). Pharyngeal pooling is detected at both pharyngeal and esophageal phases.
(MP4)

S2 Fig Moving image of case 6 of Table 4. Anterior half; nasal regurgitation is detected at pharyngeal phases before treatment of DM. Last half; the observation was disappeared after treatment with corticosteroid.
(MP4)
